# SARS-CoV-2 infections before, during, and after the Omicron wave: a 2-year Indian community cohort study

**DOI:** 10.1016/j.lansea.2024.100470

**Published:** 2024-08-21

**Authors:** Ramya Madhavan, Jackwin Sam Paul, Sudhir Babji, Isai Thamizh, Dilesh Kumar, Shainey Alokit Khakha, Aarene Rennie, Keerthana Kumar, Pavithra Dhanapal, Poornima Saravanan, Ajith Kumar, Sushil Immanuel, Vaishnavi Gandhi, Anand Kumar, Johnson John Babu, Nandu Thrithamarassery Gangadharan, Premkumar Jagadeesan, Elizabeth John, Colin Jamora, Dasaradhi Palakodeti, Rubina Bhati, Saranya Devi Thambidurai, Arati Suvatha, Anna George, Gagandeep Kang, Jacob John

**Affiliations:** a The Wellcome Trust Research Laboratory, Christian Medical College, Vellore, India; b Department of Community Health, Christian Medical College, Vellore, India; c COVID-19 Testing and INSACOG Sequencing Laboratory, Institute for Stem Cell Science and Regenerative Medicine (inStem), Bangalore, India

**Keywords:** SARS-CoV-2, Community cohort, Re-infections, Omicron, Surveillance strategies

## Abstract

**Background:**

We measured the incidence of severe acute respiratory syndrome coronavirus 2 (SARS-CoV-2) infections and re-infections in an adult community-based cohort in southern India.

**Methods:**

We conducted a 2-year follow-up on 1229 participants enrolled between May and October 2021. Participants provided vaccination histories, weekly saliva samples, and blood samples at 0, 6, 12, and 24 months. Salivary reverse transcription polymerase chain reaction (RT-PCR) and Meso-Scale Discovery panels were used for SARS-CoV-2 detection and anti-spike, anti-nucleocapsid immunoglobulin G quantification. Whole genome sequencing was performed on a subset of positive samples. SARS-CoV-2 infection incidence was measured across Pre-Omicron (May–December 2021), Omicron-I (December 2021–June 2022), and Omicron-II (July 2022–October 2023) periods.

**Findings:**

In total, 1166 (95%) participants with 83% seropositivity at baseline completed the follow-up, providing 2205 person-years of observation. Utilizing both RT-PCR and serology we identified 1306 infections and yielded an incidence rate of 591.3 per 1000 person-years (95% confidence interval, 559.6–624.3), which peaked during Omicron-I at 1418.1 per 1000 person-years (95% confidence interval, 1307.4–1535.6). During Omicron-I and II, neither prior infection nor vaccination conferred protection against infection. Overall, 74% of infections were asymptomatic.

**Interpretation:**

Integrated RT-PCR and serology revealed significant SARS-CoV-2 infection frequency, highlighting the prevalence of asymptomatic cases among previously infected or vaccinated individuals. This underscores the effectiveness of combining surveillance strategies when monitoring pandemic trends and confirms the role of non-invasive sampling in ensuring participant compliance, reflecting national transmission patterns.

**Funding:**

The study was funded by the 10.13039/100000865Bill and Melinda Gates Foundation.

**Research in context**

**Evidence before this study**

Before our investigation, there existed a significant data gap regarding severe acute respiratory syndrome coronavirus 2 (SARS-CoV-2) infections and re-infection rates in India. The two available studies from India were confined to hospital settings, which likely entail differing exposure rates. Moreover, these investigations did not monitor participants over a prolonged duration, especially in the aftermath of the Omicron wave, and lacked a community context.

**Added value of this study**

The significance of our study lies in its contribution to the current knowledge base. We conducted a detailed examination of SARS-CoV-2 infections within an Indian community cohort, both preceding and following the Omicron wave, by a 2-year intensive salivary sampling. In contrast to previous studies, our analysis included the serological fold increase across the different blood draws to identify infections that were missed by salivary RT-PCR. The study reflects the transmission dynamics, demographic characteristics, and clinical outcomes specific to this population. By utilizing non-invasive salivary samples alongside serology, our study offers an effective approach to monitoring the risk of current and future respiratory infections.

**Implications of all the available evidence**

Considering all available evidence and our findings, it becomes apparent that the experience of a small cohort in South India provides a comprehensive understanding of the impact of SARS-CoV-2 variants at a national level. Additionally, the cohort exhibited low vaccine booster uptake rates after the Omicron wave in India, emphasizing the need for newer vaccination strategies. Notably, the non-communicable disease deaths were higher in the cohort during the follow-up period, demanding careful attention and immediate interventions.

## Introduction

Re-infections with severe acute respiratory syndrome coronavirus 2 (SARS-CoV-2) have been reported since August 2020.^1^ Vaccines became available in late 2020, with neutralizing antibodies considered a correlate of protection.^2^, ^3^, ^4^ However, emerging variants were soon found to have the capacity to evade vaccine- and infection-induced immunity and cause re-infection. From late 2021, the Omicron variant and its sub-lineages have demonstrated the ability to re-infect vaccinated and previously infected individuals, with viral evolution playing a significant role in immune response evasion.^5^, ^6^, ^7^

The SIREN study, conducted in the United Kingdom (UK), monitored a cohort of healthcare professionals; it reported 7.6 re-infections and 57.3 new infections per 100 000 person-days of follow-up among individuals with and without anti-spike immunoglobulin G (IgG), respectively, before the Omicron wave. During the Omicron wave, the same cohort reported 94.2 re-infections per 1000 participants.^8^^,^^9^ Furthermore, the PHIRST-C cohort, which captured household transmission of SARS-CoV-2 in South Africa, reported a cumulative infection rate of 67.6% and 81% in their rural and urban sites, respectively, before the Omicron wave, with the majority being primary infections. Conversely, during the Omicron wave, re-infections in this cohort were higher, with primary infections contributing to only 37.9% and 28.6% of all the Omicron infections in the rural and urban sites, respectively.^10^^,^^11^

India experienced successive waves, each characterized by distinct viral lineages and varying levels of infection and severity. Dhumal et al. reported re-infection and vaccine breakthrough infection rates of 2.2% and 5.6% before the Omicron period, respectively, in a healthcare facility in India.^12^ A study among healthcare workers at a tertiary care center in Delhi during the Omicron period examined the incidence of SARS-CoV-2 infections, re-infections, outcomes, and vaccine effectiveness of two widely used vaccines against symptomatic infections. They reported an incidence of infections and re-infections of 34.8 and 45.6 per 10 000 person-days, respectively, and a vaccine effectiveness of 52.5% within 14–60 days after two doses, with a waning of vaccine protection beyond 60 days post-vaccination. Notably, these reports were restricted to healthcare settings and extended until the Omicron wave.^13^

We present a community-based cohort study conducted in Vellore, a city in southern India, focusing on the incidence of SARS-CoV-2 infections and re-infections before, during, and after the Omicron wave, in a cohort followed for 2 years. Unlike other studies conducted in India, this study provides insights into the epidemiological patterns, symptom status, and infection landscape, particularly after the Omicron wave, and provides an approach that may assist in monitoring the risk of current and future respiratory infections.

## Methods

### Study cohort and sample collection

**Fig. 1:**
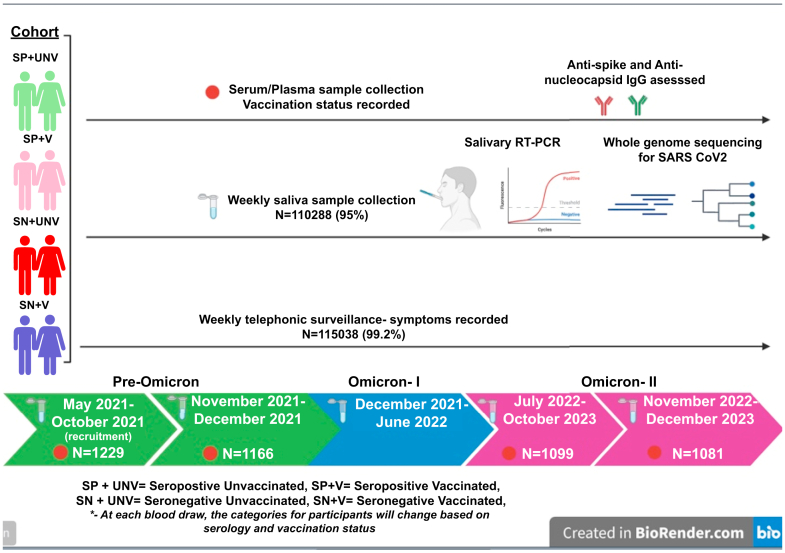
The categories, samples collected, and study procedures in the CORES cohort during the follow-up. Created in biorender.com. Ig, immunoglobulin; SARS-CoV-2, severe acute respiratory syndrome coronavirus 2.

The COVID-19 RE-infection Study (CORES) is a prospective cohort study nested within the Vellore Demographic and Health Surveillance System (VDHSS). The VDHSS monitors a population of 200,000 people across two of Vellore's four city zones. Notably, this study area has a dense homogenous population, primarily comprising individuals from lower economic backgrounds, limited educational attainment, and engaged in daily wage labor. Such demographic characteristics can potentially influence vaccination acceptance rates, healthcare service accessibility, and the likelihood of SARS-CoV-2 exposure. The study protocol has been published.^14^ Briefly, 1229 adults with no documented immunosuppression were recruited between May 2021 and October 2021 for 2 years of follow-up to capture incident SARS-CoV-2 infections and re-infections. Recruitment was initiated after the peak of the Delta wave in India, with baseline blood samples collected from all participants. Weekly saliva samples were also collected and subjected to reverse transcription polymerase chain reaction (RT-PCR) testing. A randomly selected subset of positive samples with cycle threshold (Ct) values of less than 25 underwent whole genome sequencing. In addition to the weekly saliva samples, three follow-up blood samples were collected: immediately before the Omicron wave (November 2021–December 2021), one year after recruitment (May 2022–October 2022), and 2 years after recruitment (May 2023–October 2023). These blood samples were tested for anti-spike and anti-nucleocapsid antibodies using Meso-Scale Discovery (MSD)-based panels. All RT-PCR-positive saliva and blood samples at all four time points were stored at −80 °C until further testing. Coronavirus disease 2019 (COVID-19) vaccinations were recorded at all blood draws. Fig. 1 illustrates the samples, procedures and various categories of individuals recruited and monitored in the CORES study. This study was approved by the Institutional Review Board of Christian Medical College, Vellore (IRB min no: 13585) and adhered to the Strengthening the Reporting of Observational Studies in Epidemiology (STROBE) guidelines. Written informed consent was obtained from all participants.

### Definitions

Participants were categorized as seropositive vaccinated (SP + V)/seropositive unvaccinated (SP + UNV) if their anti-spike IgG concentration measured was 1960 AU/mL or higher by electrochemiluminescence immunoassay using the MSD platform based on their history of vaccination with or without at least one dose of any COVID-19 vaccine. Similarly, seronegative vaccinated (SN + V)/seronegative unvaccinated (SN + UNV) participants were classified based on an anti-spike IgG concentration of less than 1960 AU/mL and vaccination history. The participant categories were dynamic, with changes occurring in serostatus or vaccination history over time.

Single infection was defined as:

Only one incident infection in SP + V, SN + V, and SN + UNV.

Re-infection was defined as:

i) Any incident infection recorded in the SP + UNV group during the follow-up, as seropositivity was considered as a prior exposure. If more than one incident infection was recorded, it was considered as a second/third re-infection with an interval between the events of more than 90 days.
ii) More than one incident infection in SP + V, SN + V, and SN + UNV, with an interval between the two events of more than 90 days.

### Laboratory procedures

#### Salivary RT-PCR

Salivary samples were collected weekly and transported to the laboratory within a mean (standard deviation) of 2.2 (1.5) hour. RT-PCR was performed targeting the E, N, and RdRp genes using a CoviDx mPlex-4R SARS-CoV-2 RT-PCR Detection Kit (NeoDx Ltd, Bengaluru, Karnataka, India).^15^ These three targets were used for RT-PCR since the spike gene frequently mutates, posing a challenge for SARS-CoV-2 detection. Five samples were pooled in a well; any one gene target with a cycle threshold (Ct) of less than 37 was considered positive, as per the kit instructions. If any pool was positive, individual samples were tested. We adopted systematic random sampling and selected every 5th sample for whole genome sequencing after generating the list of samples that had a ct value of less than 25. We followed the ARTIC protocol (Illumina United States) for whole genome sequencing.^16^ The number of samples sequenced is described in Supplementary Table S1.

#### Sequencing and phylogenetic analysis

Sequencing libraries were prepared using the COVIDSeq library preparation kit following manufacturer's guidelines (Illumina, United States). Libraries were diluted to 8 pmol and spiked with PhiX sequencing control. Next-generation sequencing was conducted on an MiSeq sequencing platform (Illumina, United States) using the MiSeq Reagent Kit v3 for 600 cycles with a 300 bp paired-end reads method. Sample reads were assembled using the Illumina COVIDSeq Test Pipeline, which performs variant calling and generates sequences using the FASTA format. The sequences for all the study strains were deposited to Global Initiative on Sharing All Influenza Data (GISAID) under accession numbers as listed in Supplementary Table S2. A preliminary analysis of the sequences was performed on the Nextclade web platform.^17^ SARS-CoV-2 lineages (or clades) were assigned, and mutations were marked, taking Wuhan-Hu-1/2019 (MN908947) as a reference.^18^ For phylogenetic analysis, high-quality nucleotide sequences from different lineages were downloaded from the GISAID database as a reference.^18^ The Wuhan-Hu-1 genome (NC_045512) sequence was included for the analysis. The final analysis dataset (117 study sequences + 561 reference sequences) was aligned using the Nextclade webserver. The Model Finder program was used to identify the optimal substitution model that best fit the sequence datasets using the Bayesian information criterion.^19^ Maximum likelihood trees were constructed using IQTree-2 software with 1000 bootstrap replicates.^20^ The inferred tree was visualized and annotated using the Interactive Tree Of Life web server.^21^

### Antibody assay

#### MSD-based assay

The MSD-based multiplex electrochemiluminescence assay was employed for the simultaneous detection of a broader repertoire of IgG antibodies against the SARS-CoV-2 spike (S), spike receptor-binding domain, spike N terminal domain, and nucleocapsid antigen using the V-PLEX SARS-CoV-2 Panel 1 kit according to the manufacturer's protocol at baseline, Pre-Omicron, and 12 and 24 months of follow up.^22^ It is a quantitative assay with early sensitivity (Day 0–14 of infection) of 84%, late sensitivity (Day 15+ of infection) of 98% and specificity of 99%. All samples were tested at an initial dilution of 1:5000. Any samples that were above or below the range of detection were retested in lower (1:1000) or higher dilutions (up to 1:50000), and the results were expressed as AU/mL. The two- and four-fold increase in anti-spike and anti-nucleocapsid were used in the incidence calculation in the SP + V/UNV and SN + V/UNV groups, respectively.

#### Assessing the incidence of SARS-CoV-2 infection

The incidence of SARS-CoV-2 infection was calculated during the Pre-Omicron (May 19, 2021–December 20, 2021), Omicron-I (December 21, 2021–June 6, 2022), and Omicron-II (June 7, 2022–October 31, 2023) periods within the four categories. We have chosen the Omicron periods based on the six monthly blood draws within the cohort, which fortuitously allowed for a pre- and post-Omicron antibody profiling. The recruitment and pre-Omicron samples established the serostatus of the cohort prior to the emergence of the Omicron variant in India. The 12th month blood draw reflected the level of exposure to the Omicron variant (BA.1, BA.2) and the 24th month blood draw reflected the exposure due to the other Omicron sub-lineages (BA.2.75, BA.5, XBB.1.16). The Omicron-I period (December 2021–June 6, 2022) and the Omicron II period (June 2022–October 31, 2022) were due to different Omicron lineages identified by genomic sequencing. Since the incidence could potentially be underestimated based on salivary RT-PCR alone due to its lower sensitivity, we also examined the fold increase in anti-spike and anti-nucleocapsid antibody levels. Therefore, we analyzed the incidence among the four groups using different criteria with three possible definitions. These encompassed a positive RT-PCR result alone (Definition 1), either a positive RT-PCR result and/or fold increase in anti-spike (MSD) anti-nucleocapsid IgG antibody levels in the unvaccinated alone (Definition 2), and either a positive RT-PCR result and/or fold increase in anti-nucleocapsid or anti-spike antibodies in both vaccinated and unvaccinated individuals (Definition 3), as outlined in Table 1. Definition 3 was adopted for subsequent analysis as we were able to capture the infections that were missed by Definitions 1 and 2.

**Table 1:** Definitions for the calculation of incidence rates based on RT-PCR positivity and/or serological fold increase in the four categories of, seropositive and unvaccinated [SP + UNV], and seropositive and vaccinated [SP + V], seronegative and unvaccinated [SN+UNV], seronegative and vaccinated [SN+V].

Definition 1	Incidence rates were calculated based on positive RT-PCR results only
Definition 2	Incidence rates across the four categories were determined based on the following criteria: i] SP + UNV—Either a positive RT-PCR result and/or a two-fold increase in anti-spike or anti-nucleocapsid levels ii] [SP + V/SN + V]—A positive RT-PCR result only iii] SN + UNV—A positive RT-PCR result or a four-fold increase in anti-spike or anti-nucleocapsid levels
Definition 3	The incidence rates across the four categories were determined based on the criteria outlined below: i] SP + UNV—A positive RT-PCR result or a two-fold increase in anti-spike or anti-nucleocapsid levelsii] SP + V [Covishield]^a^—A positive RT-PCR result or a two-fold increase in anti-nucleocapsid levels iii] SN + V [Covishield]^a^—A positive RT-PCR result or a four-fold increase in anti-nucleocapsid levels iv] SP + V [Covaxin]^b^—A positive RT-PCR result only v] SN + V [Covaxin]^b^—A positive RT-PCR result only vi] SN + UNV—A positive RT-PCR result or a four-fold increase in anti-spike or anti-nucleocapsid levels

RT-PCR: reverse transcription polymerase chain reaction; SN: seronegative; SP: seropositive; UNV: unvaccinated; V: vaccinated.

a In addition to the above-mentioned definitions, the anti-spike IgG fold increase was assessed only when there was no vaccination between blood draws.

b In addition to the above-mentioned definitions, the anti-spike IgG/anti-nucleocapsid IgG fold increase was assessed when there was no vaccination between blood draws.

Concerning the application of serology to detect infections, it is important to highlight that by March 2021, India had granted authorization for the emergency use of two domestically produced vaccines, which had been made available to the wider population. These were the Serum Institute of India's recombinant non-replicating adenoviral vectored vaccine, ChAdOx-nCov-19 (Covishield), and Bharat Biotech's whole virion inactivated vaccine, BBV152 (Covaxin), which incorporates a TLR7/8 agonist and alum as adjuvants. As BBV152 constitutes a whole virion-inactivated vaccine, natural infection and vaccine-induced responses in vaccinated individuals cannot be distinguished on the basis of increased levels of anti-nucleocapsid antibodies. Consequently, the estimated incidence of infection in these participants was solely based on RT-PCR + results when there was a vaccination between the two blood draws, as defined by Definition 3.

### Statistical analyses

The incidence analysis was restricted to the 1166 individuals who completed 2 years of follow-up. We initially accounted for a 10% dropout rate during the sample size calculation at the study's outset. Despite some samples being missed in the 2nd-4th blood draw, we included those individuals in the incidence rate calculation. The time contributed to the study until the participant was censored has been added in the person-time analysis. The incidence of SARS-CoV-2 infections was calculated based on RT-PCR and/or a fold increase in anti-spike or anti-nucleocapsid IgG based on the MSD platform. Participants were classified based on their serology and vaccination status prior to each period and therefore they switched categories between the periods. The incidence rates in each category are reported per 1000 person-years of observation. The Chi-square test was used to compare the demographic characteristics and symptom assessment among the no infection, single infection, and re-infection groups for categorical variables and Analysis of Variance (ANOVA) was used for continuous variables. Risk Ratios were calculated using a logistic regression model with infection as an outcome among seronegative individuals. The seropositive individuals were used as the reference and adjusted for vaccination status. A similar analysis was conducted for unvaccinated individuals, adjusting for serology status. The Sankey plot was constructed using the network D3 package of R software to illustrate the dynamics of serostatus and vaccination status within the cohort.^23^ All statistical analyses were performed using R software (version 4.2.2, R Foundation for Statistical Computing, Vienna, Austria). All statistical tests were two-sided with a significance level of less than 0.05.

### Ethical statement

The study has been approved by the Institutional Review Board of Christian Medical College, Vellore. Written informed consent was obtained from all participants.

### Role of the funding source

The funders have no role in the study design, data collection, analysis, and interpretation of the data.

## Results

### Cohort follow-up

Of the 1229 individuals recruited, 1166 completed the 2-year study, resulting in 2205.2 person-years of observation, with 115,038 (99.8%) weekly telephonic surveillance contacts. The reasons for participant dropout were migration, refusal to provide samples, and non-COVID-19-related deaths (Supplementary Table S3). Of the 115,288 expected saliva samples, 110,288 (95.7%) samples were collected and underwent RT-PCR analysis. Regarding blood samples, 1229, 1166 (94%), 1101 (89%), and 1081 (87%) were collected at enrollment/baseline and at 5–6, 12, and 24 months of follow-up, respectively. Missing samples were attributed to migration, refusal, or temporary unavailability. The baseline demographic characteristics of the cohort are described in Table 2.

**Table 2:** Baseline demographic characteristics of the CORES cohort with infections and re-infections during the study follow-up [n = 1166].

	No infection	Single Infection	Re-infection	Total Participants	p-value
**Age**					
Median [Range]	41.6 [19–75]	43.5 [19–81]	45.4 [18–82]	43.8 [18–82]	
SD	12.2	12.4	12.9	12.6	
**Age group**					
18–30 years	44 [26.3%]	68 [40.7%]	55 [32.9%]	167	0.002
31–45 years	123 [24%]	191 [37.2%]	199 [38.8%]	513	
46–60 years	69 [18.9%]	155 [42.5%]	141 [38.6%]	365	
Above 60 years	17 [14%]	37 [30.6%]	67 [55.4%]	121	
**Gender:**					
Male	118 [20.2%]	217 [37.2%]	248 [42.5%]	583	0.1
Female	135 [23.2%]	234 [40.1%]	214 [36.7%]	583	
**Co-Morbid illness**^a^					
No	195 [22.4%]	338 [38.9%]	336 [38.7%]	869	0.4
Yes	58 [19.5%]	113 [38%]	126 [42.4%]	297	
**Co-Morbidities details:**					
**Diabetes**					
No	217 [22%]	383 [38.8%]	388 [39.3%]	988	0.8
Yes	36 [20.2%]	68 [38.2%]	74 [41.6%]	178	
**Hypertension**					
No	219 [22%]	382 [38.4%]	393 [39.5%]	994	0.8
Yes	34 [19.8%]	69 [40.1%]	69 [40.1%]	172	
**Heart disease**					
No	253 [21.9%]	444 [38.4%]	459 [39.7%]	1156	0.08
Yes	0 [0%]	7 [70%]	3 [30%]	10	
**COPD**					
No	253 [21.7%]	450 [38.6%]	462 [39.7%]	1165	0.5
Yes	0 [0%]	1 [100%]	0 [0%]	1	
**CVA**					
No	250 [21.6%]	450 [38.8%]	460 [39.7%]	1160	0.2
Yes	3 [50%]	1 [16.7%]	2 [33.3%]	6	
**History of COVID-19 before enrolment**					
Yes	6 [23.1%]	13 [50%]	7 [26.9%]	26	0.4
No	247 [21.7%]	438 [38.4%]	455 [39.9%]	1140	
**Occupation**^b^**:**					
Professional	0 [0%]	1 [50%]	1 [50%]	2	0.4
Semi Professional	3 [14.3%]	11 [52.4%]	7 [33.3%]	21	
Clerk/Shop/Farm	10 [11.8%]	32 [37.6%]	43 [50.6%]	85	
Skilled worker	61 [22.1%]	109 [39.5%]	106 [38.4%]	276	
Semi-Skilled worker	18 [18.4%]	39 [39.8%]	41 [41.8%]	98	
Unskilled worker	45 [25.4%]	60 [33.9%]	72 [40.7%]	177	
Unemployed	116 [22.9%]	199 [39.3%]	192 [37.9%]	507	
**Family income:**					
Median [Range]	6000 [1000–45,000]	6000 [1000–60,000]	6000 [1000–65,000]	6000 [1000–65,000]	
**Total no. of family members**					
Median [Range]	5 [1–12]	4 [1–13]	4 [2–14]	4 [1–14]	
**Vaccination status**					
No	214 [23.1%]	342 [36.9%]	370 [40%]	926	0.02
Yes	39 [16.3%]	109 [45.4%]	92 [38.3%]	240	
**Vaccinated participant's details [n** = **240]:**					
**Vaccine type**					
Covaxin	9 [18.8%]	25 [52.1%]	14 [29.2%]	48	0.3
Covishield	30 [15.6%]	84 [43.8%]	78 [40.6%]	192	
**Vaccine dosage**					
1 Dose	32 [17.6%]	80 [44%]	70 [38.5%]	182	0.07
2 Doses	7 [12.1%]	29 [50%]	22 [37.9%]	58	

COPD: chronic obstructive pulmonary disease; COVID-19: coronavirus disease 2019.

a Comorbidities were reported by the participants at the time of recruitment.

b Occupation was a direct correlate of access to healthcare.

**Fig. 2:**
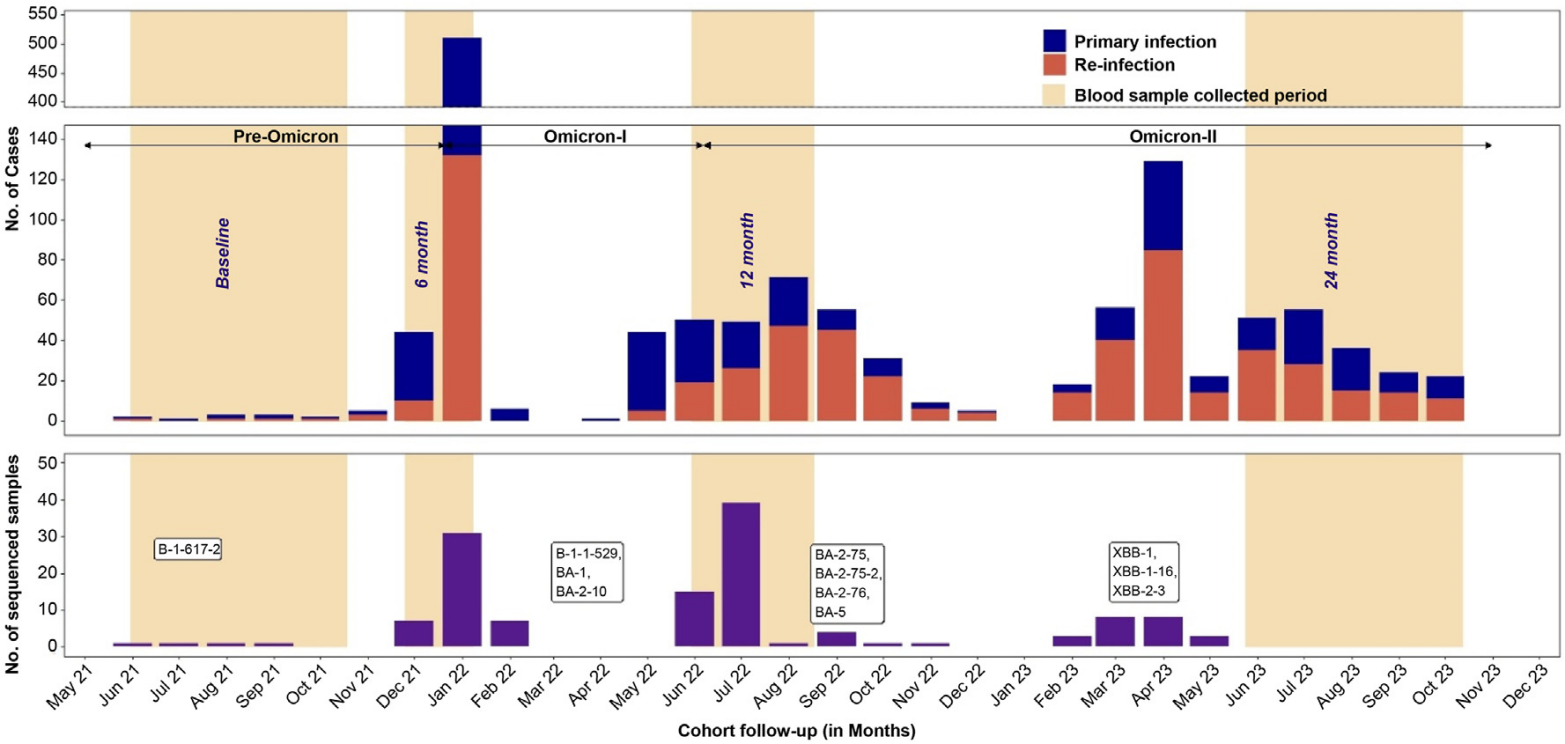
Pattern of SARS-CoV-2 primary and re-infections in the CORES cohort, along with the variants identified through sequencing during follow-up. Primary infection was defined as one incident infection in SP + V, SN + V, and SN + UNV and re-infection was defined as any incident infection recorded in the SP + UNV group during the follow-up as seropositivity was considered as a prior exposure. If more than one incident infection was recorded, it was considered as a second/third re-infection with an interval between the events of more than 90 days.

Fig. 2 illustrates the pattern of infections that were captured by RT-PCR and serology (Definition 3) during the follow-up period. The cohort experienced a total of 1304 incident infections, with 51 infections (38 primary infections and 13 re-infections) occurring during the Pre-Omicron period, 606 (455 primary infections and 151 re-infections) during the Omicron-I, and 647 (232 primary infections and 415 re-infections) during the Omicron-II period (Fig. 2).

**Fig. 3:**
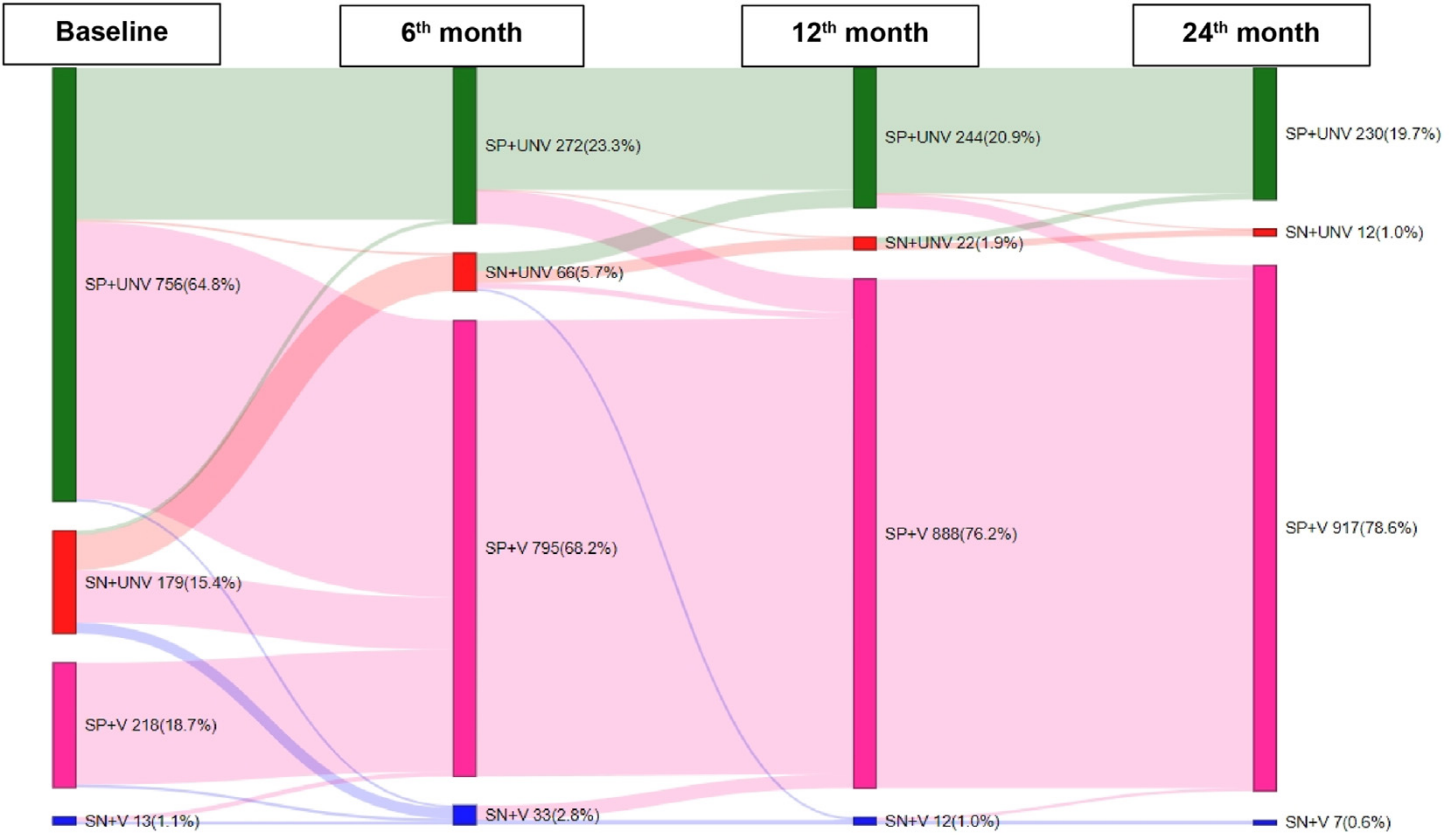
Sankey plot demonstrating serology and vaccination status dynamics over 2 years of follow-up in the CORES cohort. SN: seronegative; SP: seropositive; UNV: unvaccinated; V: vaccinated.

In Fig. 3, the Sankey plot depicts the dynamic changes in serology and vaccination status over the 2-year follow-up period within the cohort. At the time of recruitment, 83% of the cohort demonstrated antibodies against the SARS-CoV-2 spike protein, with 19.8% having received at least one dose of either the adenoviral vectored vaccine ChAdOx-nCoV-19 (Covishield) or whole virion-inactivated vaccine BBV152 (Covaxin). This high antibody prevalence reflected the substantial levels of infection following the Delta wave that occurred in India between March and May 2021. During the second blood sampling, conducted just before the Omicron wave, 91% of participants were found to be seropositive, and 69% reported receiving at least one vaccine dose.

Approximately 4–6 months after the Omicron wave, at the one-year follow up, the vaccination rate had increased to 76%, with 96% of participants being seropositive. At the 2-year follow-up, 78% had received at least one vaccine dose, and 98% were seropositive. Notably, among the unvaccinated cohort, 12 (1%) participants showed no seroconversion over the 2-year period. Within the vaccinated group, 0.6% (7/1166) displayed no seroconversion despite documented infections by RT-PCR in both groups (Fig. 3). The vaccination status and types of vaccines received by the cohort participants are described in Supplementary Table S4.

**Fig. 4:**
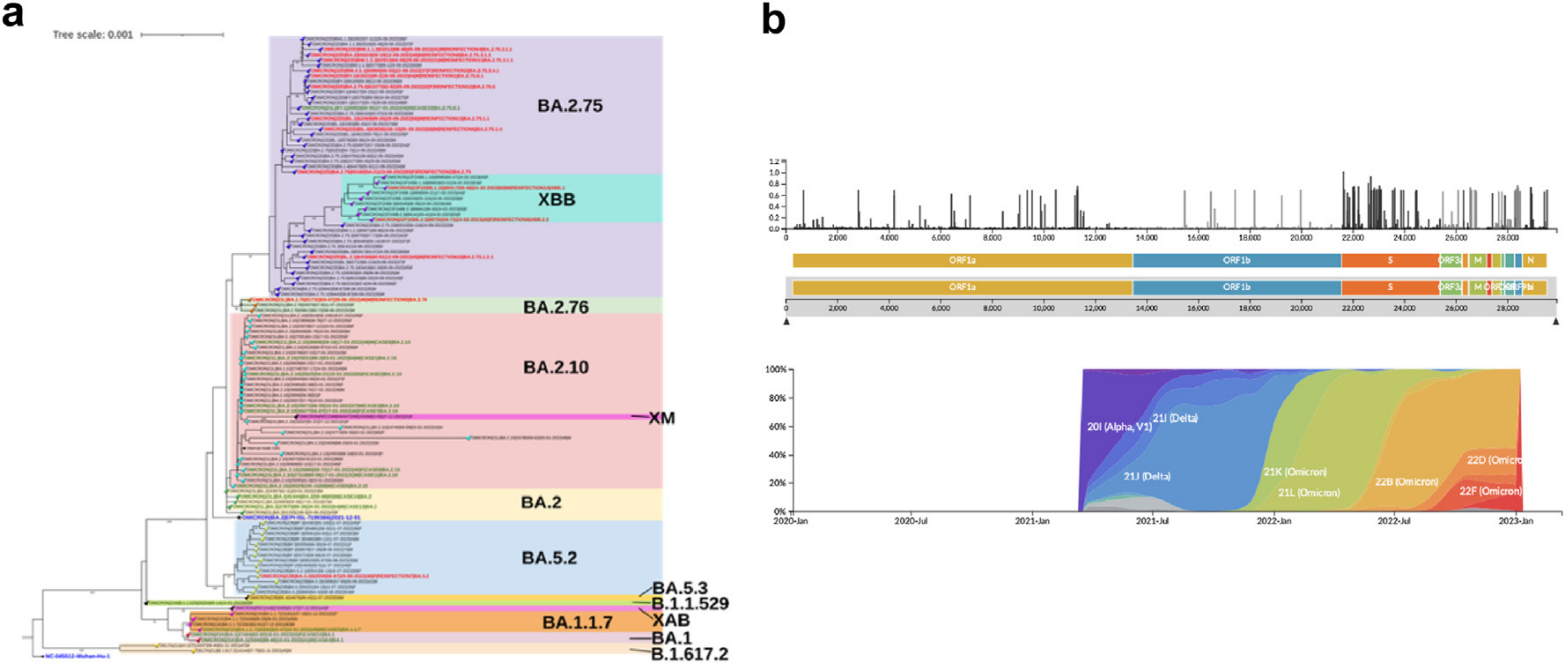
Whole genome sequencing of RT-PCR positive salivary samples within the cohort. a) Maximum likelihood tree of the CORES study samples compared with the Wuhan and BA.2 reference genomes, constructed using IQTree software. The green text indicates primary infection, and the red text indicates re-infection detected by RT-PCR. b) Comparison of the genomes submitted to GISAID during the same study period from Southeast Asia.

**Fig. 5:**
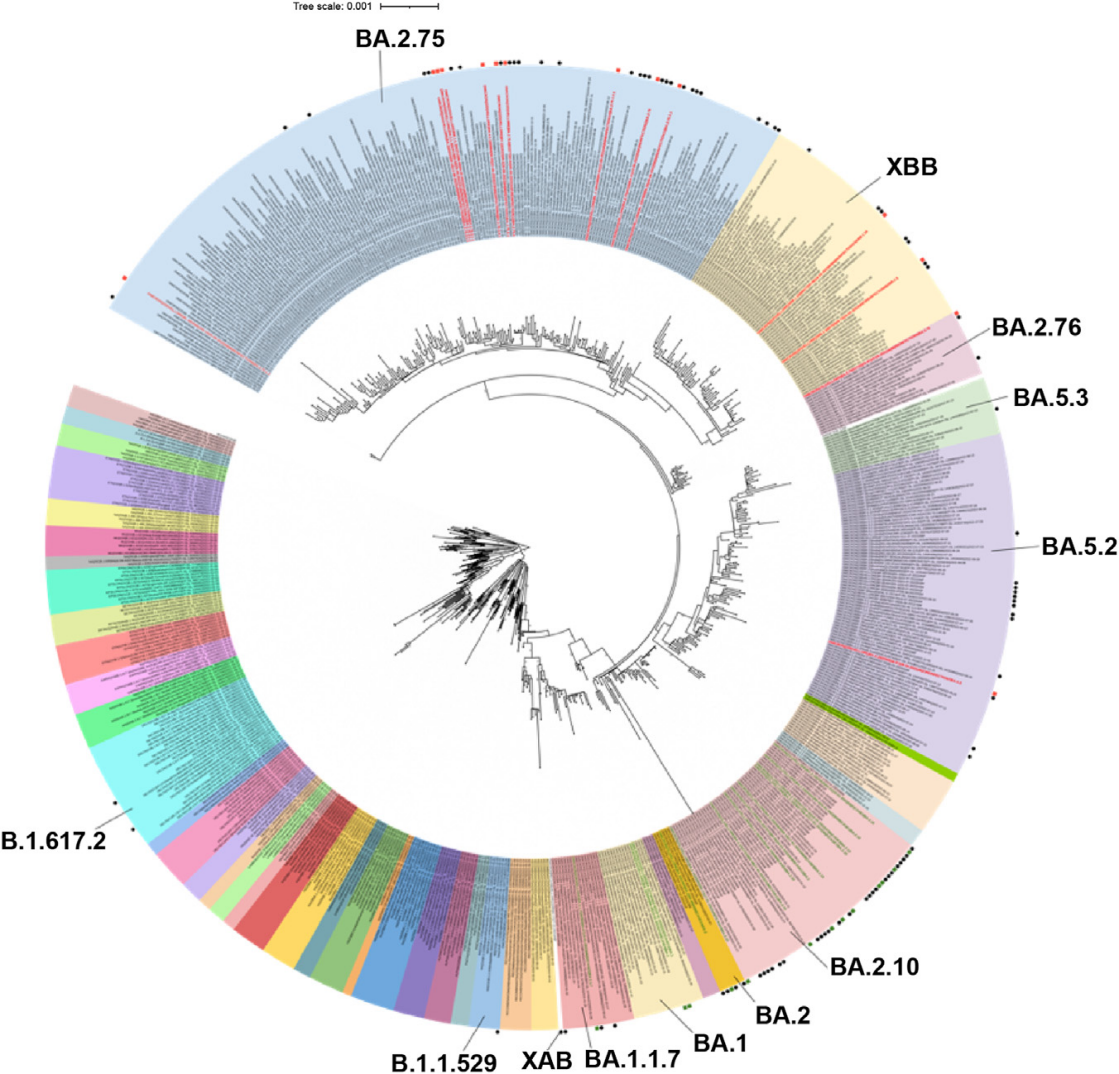
Comparison of the study samples with the 561 representative reference genomes selected globally and from various states in India. Black dots symbolize the study samples, green dots denote the primary infection, and red dots signify re-infection.

Among the 993 SARS-CoV-2-positive samples identified by RT-PCR, 151, exhibiting a Ct value less than 25, were selected for whole genome sequencing. Of these 151 samples, 1 was excluded due to an ID mismatch, 11 failed sequencing, and 22 exhibited low coverage, as illustrated in Supplementary Table 1. Among the 117 successfully sequenced samples, 14 were from participants for whom both the first and second RT-PCR positive samples were sequenced. As depicted in Fig. 2, the identified lineages during the Pre-Omicron period were Delta (B.1.617.2), while those during the Omicron-I period were Omicron (B.1.1.529) and its sub-lineages (BA.1, BA.2, BA.2.10). Throughout the Omicron-II period, the assigned lineages included BA.5, BA.2.75, BA.2.76, XM, XBB.1.16, XBB.2.3, and XAB. Among those re-infected during the study, the lineage distribution was BA.2.75 (10/14), BA.2.76 (1/14), BA.5.2 (1/14), XBB.1.16 (1/14), and XBB.2.3 (1/14). Phylogenetic analysis, utilizing Wuhan-Hu-1 and BA.2 as reference sequences, revealed clustering, with the lineage distribution within the cohort temporally aligned with circulating strains in Southeast Asia,^24^ as shown in Fig. 4. Further, a minimum spanning tree with 561 global and Indian genomes from GISAID demonstrated similar clustering (Fig. 5), with the XBB.1.16 sub-lineage separated within the XBB lineage.

### Identification of SARS-CoV-2 infections

Table 3 demonstrates the incidence of SARS-CoV-2 infections in the 1166 participants according to the three definitions for detecting SARS-CoV-2 infection described in the Methods section. The overall incidence was 452, 573, or 591 during 2205 person-years of observation as per Definitions 1, 2, and 3, respectively. Definition 3 was used for further analysis of the incidence of infection in the four categories of participants as it helped capture asymptomatic infections missed by salivary RT-PCR (Table 3). Although the incidence was relatively lower in the SP + UNV/V category than in that of SN + UNV/V during the pre-Omicron period, the pattern of incidence rates was similar across all the categories during the Omicron-I and Omicron-II periods. Among all three time periods, the Omicron-I period was found to show the highest incidence across all categories (Table 3) (Supplementary Table S5).

**Table 3:** Incidence rates within the four categories based on different definitions.

Definition	SP + UNV	SP + V^a^	SN + UNV	SN + V^a^	Overall
**Definition 1** **Based on RT-PCR alone**					**452.6 [424.9–481.54]**
Pre-Omicron	34.2 [12.6–74.5]	9.85 [1.2–35.6]	69.8 [14.4–203.9]	181.8 [59–424.3]	
Omicron-I	1490.1 [1232.3–1785.8]	1108 [994.8–1230.5]	1928.2 [1327.3–2707.9]	1436.1 [920.1–2136.8]	
Omicron-II	328.1 [264.5–402.4]	351.8 [316.1–390.5]	358.8 [164–681]	248.9 [80.8–580.9]	
**Definition 2** **Based on RT-PCR and serology in the unvaccinated**					**573.2 [542**–**605.7]**
Pre-Omicron	74.19 [39.5–126.9]	39.38 [17–77.6]	209.27 [95.7–397.3]	218.2 [80.1–474.9]	
Omicron-I	1630.1 [1360–1938.2]	1263.5 [1142.4–1394]	1986.6 [1375.8–2776.1]	1436.1 [920.1–2136.8]	
Omicron-II	466.2 [389.8–553.2]	497.1 [454.5–542.7]	366.9 [167.8–696.4]	253.8 [82.4–592.3]	
**Definition 3** **Based on RT-PCR and serology in all participants**					**591.3 [559.6**–**624.3]**
Pre-Omicron	74.19 [39.5–126.9]	113.22 [71.8–169.9]	209.2 [95.7–397.3]	218.1 [80.1–474.9]	
Omicron-I	1630.2 [1360–1938.3]	1333.4 [1208.9–1467.2]	1986.6 [1375.8–2776.1]	1436.1 [920.1–2136.8]	
Omicron-II	466.2 [389.8–553.2]	500.1 [457.3–545.8]	366.9 [167.7–696.5]	253.7 [82.4–592.3]	

Pre-Omicron: May 2021–December 20, 2021; Omicron-I: December 21, 2021–June 2022; Omicron-II: July 2022–October 2023.

Participants were classified during each period based on their serology and vaccination status. All the analysis is based on person-years of observation, and the results are tabulated as incidence per 1000 person-years of observation.

RT-PCR: reverse transcription polymerase chain reaction; SN: seronegative; SP: seropositive; UNV: unvaccinated; V: vaccinated.

a The vaccinated category includes all individuals who had taken at least one dose of vaccine.

### Risk of SARS-CoV-2 infections among seronegative individuals

We assessed the risk of SARS-CoV-2 infection among seronegative individuals compared to seropositive individuals during the Pre-Omicron, Omicron-I, and Omicron-II periods by calculating the risk ratio (RR). During the Pre-Omicron period, seronegative individuals exhibited an RR (95% CI) of 2.24 (1.19–4.00), indicating a two-fold higher risk compared with their seropositive counterparts. Conversely, during the Omicron-I period, the RR (95% CI) was 1.24 (0.90–1.60), illustrating a diminished risk differential. During the Omicron-II period, the RR for seronegative individuals further decreased to 0.64 (0.36–1.05), suggesting a lower risk of SARS-CoV-2 infection compared with seropositive individuals. Importantly, the risk pattern remained consistent after adjusting for vaccination status, age, gender throughout all three periods, as indicated in Table 4, Supplementary Table S6.

**Table 4:** Risk ratio among seronegative individuals compared to seropositive individuals across various periods to assess the risk of SARS-CoV-2 infection.

Time periods	Serology status	Total cases	PYO	Incidence rate [95% CI]	Relative risk [95% CI]	Adjusted RR [95% CI]
Pre-Omicron	Negative	15	70.51	212.74 [119.07–350.89]	2.24 [1.19–4]	2.34 [1.24–4.21]
	Positive	36	378.37	95.14 [66.64–131.72]	ref	Ref
Omicron-I	Negative	58	33.83	1714.65 [1302.01–2216.58]	1.23 [0.93–1.59]	1.15 [0.87–1.5]
	Positive	548	393.51	1392.58 [1278.41–1514.21]	ref	ref
Omicron-II	Negative	14	44.23	316.51 [173.04–531.05]	0.64 [0.36–1.05]	0.65 [0.37–1.07]
	Positive	633	1284.74	492.71 [455.07–532.63]	ref	ref

Adjusted RR- The risk ratio is adjusted for the vaccination status of the individuals.

CI: confidence interval; PYO: person-years of observation; RR: relative risk.

Participants were classified during each period based on their serology status.

### Risk of SARS-CoV-2 infection among unvaccinated individuals

Unvaccinated individuals exhibited an RR (95% CI) of 0.8 (0.4–1.4), 1.3 (1.1–1.6), and 0.9 (0.7–1.1) compared to those of vaccinated individuals during the Pre-Omicron, Omicron-I, and Omicron-II periods, respectively. Adjusting for serological status, age and gender did not alter the RR pattern (Table 5), Supplementary Table S7.

**Table 5:** Risk ratio for unvaccinated individuals compared to vaccinated individuals across different periods to assess the risk of SARS-CoV-2 infection.

Time periods	Vaccination	Total cases	PYO	Incidence rate [95% CI]	Relative risk [95% CI]	Adjusted RR [95% CI]
Pre-Omicron	Unvaccinated	22	218.23	100.8 [63.18–152.63]	0.8 [0.5–1.4]	0.7 [0.4–1.3]
	Vaccinated	29	230.65	125.7 [84.2–180.6]	ref	ref
Omicron-I	Unvaccinated	162	95.63	1693.9 [1443.2–1975.9]	1.3 [1.1–1.5]	1.2 [1–1.5]
	Vaccinated	444	331.71	1338.5 [1216.9–1469]	ref	ref
Omicron-II	Unvaccinated	140	305.51	458.2 [385.5–540.8]	0.9 [0.8–1.1]	0.9 [0.8–1.1]
	Vaccinated	507	1023.47	495.4 [453.2–540.4]	ref	ref

Adjusted RR- The risk ratio is adjusted for the serology status of the individuals.

CI: confidence interval; PYO: person-years of observation; RR: relative risk.

Participants were classified during each period based on their vaccination status.

### Status of symptoms following single and re-infections with SARS-CoV-2

We have recorded the symptoms every week just before sample collection and also immediately after the participant tested positive by RT-PCR. Among the 1306 SARS-CoV-2 infections recorded, 998 (76%) were asymptomatic and 306 (24%) were symptomatic. Among the 306 (24%) symptomatic episodes, 178 (58.2%) were single infections and 128 (41.8%) were re-infections (Table 6). No participant required hospitalization or intensive care. Incidence rates were comparable between the categories among the symptomatic individuals (Supplementary Table S8).

**Table 6:** Comparison of symptom status between single infection and re-infected participants.

	Single infection	Re-infection	Total infections	p-value
**Asymptomatic**	547 [75.4%]	451 [77.9%]	998 [76.5%]	0.30
**Symptomatic**	178 [24.6%]	128 [22.1%]	306 [23.5%]	
**Symptom details: [n** = **306]**				
**Fever**				
No	115 [64.6%]	80 [62.5%]	195 [63.7%]	0.80
Yes	63 [35.4%]	48 [37.5%]	111 [36.3%]	
**Cough**				
No	128 [71.9%]	87 [68%]	215 [70.3%]	0.50
Yes	50 [28.1%]	41 [32%]	91 [29.7%]	
**Loss of smell**				
No	177 [99.4%]	127 [99.2%]	304 [99.3%]	1.0
Yes	1 [0.6%]	1 [0.8%]	2 [0.7%]	
**Difficulty in breathing**				
No	177 [99.4%]	122 [95.3%]	299 [97.7%]	0.05
Yes	1 [0.6%]	6 [4.7%]	7 [2.3%]	
**Other symptoms**				
No	35 [19.7%]	29 [22.7%]	64 [20.9%]	0.60
Yes	143 [80.3%]	99 [77.3%]	242 [79.1%]	
**Other symptoms details: [n** = **242]**				
**Cold**				
No	52 [36.4%]	36 [36.4%]	88 [28.8%]	1.0
Yes	91 [63.6%]	63 [63.6%]	154 [50.3%]	
**Tiredness**				
No	140 [97.9%]	97 [98%]	237 [77.5%]	1.0
Yes	3 [2.1%]	2 [2%]	5 [1.6%]	
**Stomach pain**				
No	138 [96.5%]	97 [98%]	235 [76.8%]	0.8
Yes	5 [3.5%]	2 [2%]	7 [2.3%]	
**Headache**				
No	130 [90.9%]	89 [89.9%]	219 [71.6%]	1.0
Yes	13 [9.1%]	10 [10.1%]	23 [7.5%]	
**Throat pain**				
No	136 [95.1%]	95 [96%]	231 [75.5%]	1.0
Yes	7 [4.9%]	4 [4%]	11 [3.6%]	
**Chest pain**				
No	143 [100%]	97 [98%]	240 [78.4%]	0.3
Yes	0 [0%]	2 [2%]	2 [0.7%]	
**Body pain [includes leg pain/neck pain/back pain/hand pain]**				
No	101 [70.6%]	71 [71.7%]	172 [56.2%]	1.0
Yes	42 [29.4%]	28 [28.3%]	70 [22.9%]	

## Discussion

We examined the rates of SARS-CoV-2 infection in a community-based cohort in south India before, during, and after the Omicron wave over a 2-year period and identified 1306 incident cases of SARS-CoV-2 infections, which reflected the pattern of infection from national reporting. Notably, the combined approach of salivary sampling and serology facilitated the detection of asymptomatic SARS-CoV-2 infections. This type of data can assist in developing testing strategies, enhancing contact tracing efforts, and implementing targeted surveillance measures to control the spread of future respiratory viral infections with pandemic potential.

Salivary samples collected for SARS-CoV-2 surveillance were effective in maintaining high compliance and measuring infection rates, with pooled salivary sample testing proving to be an efficient method with quick results. All laboratory testing of infections based on periodic sample collection has the potential to miss incident infections, when the periods of virus shedding are short and the amount of virus being shed is variable. We developed a set of definitions that utilizes data generated by salivary PCR as well as repeated serology and history of infection or vaccination to determine the incidence of SARS-CoV-2 infection within the cohort The use of a composite definition allowed for analysis of the relative value of separate and combined testing. The incident infections captured by salivary RT-PCR alone were 993 vs.1306 infections, which were determined by combining serology data with salivary RT-PCR. Thus, 313 (23.9%) incident infections that would have been missed by salivary RT-PCR testing alone were identified by serology. This suggests the utility of integrated surveillance strategies for future cohort studies to capture true infection rates.

We observed the highest incidence of infections during the Omicron-I period compared with the Pre-Omicron and Omicron-II periods. The increased transmissibility of the Omicron variant, in comparison to prior variants and other Omicron sub-lineages, along with its capacity to evade vaccine-induced immunity, played a significant role.^25^^,^^26^ This is consistent with other cohort studies performed in South Africa, Qatar, and the UK, where infections due to Omicron, BA.1, and BA.2 were found to be higher than those caused by pre-Omicron variants.^11^^,^^27^^,^^28^

Seronegative individuals had 2.34 times higher risk of acquiring SARS-CoV-2 infection as compared to seropositive participants after adjusting for vaccination status during the pre-Omicron period (aRR (95% CI), 2.34 (1.24–4.21)). In contrast, no difference in risk was observed during the Omicron-I and Omicron-II periods. Similar observations were seen among unvaccinated individuals who had modest protection during the Omicron-I and Omicron-II periods compared to vaccinated individuals after adjusting for the serostatus (aRR (95% CI), 1.2 (1–1.5) during Omicron-I and 0.9 (0.8–1.1) during Omicron-II period among unvaccinated individuals compared to vaccinated individuals). These findings align with findings from the SIREN cohort, which reported a crude incidence rate of 2.38 per 10,000 during Delta wave and 33.51 per 10,000 during the Omicron wave among prior infected (seropositive to SARS-CoV-2 spike) individuals. Similarly, following two-dose mRNA vaccination (BNT162b2), their crude incidence rate was 9.15 per 10,000 during the Delta wave and 73.96 per 10,000 during the Omicron wave.^28^ During the Omicron-II period, nearly ∼99% of the sampled population in the cohort had anti-spike antibodies to SARS-CoV-2 with only 1% remaining seronegative. This observation is consistent with results from a South African cohort study, where a comparable shift in the immunological landscape was noted following the Omicron wave, attributed to numerous infections and vaccination efforts, with just 5.6% of the population not exposed to SARS-CoV-2.^11^

A meta-analysis found that re-infections during and after the Omicron wave exhibited a lower risk of symptomatic infection and severe disease compared with primary infections, with most cases being mild.^29^ Similarly, we observed cases of re-infection with symptoms in the present study; however, none necessitated medical attention or hospitalization. Additionally, our study recorded a marginally higher mortality rate than anticipated (22/1166, 1%), though these deaths were not directly attributable to COVID-19. This implies that non-communicable diseases emerged as a considerable public health concern amidst the pandemic, warranting meticulous attention and intervention.^30^

Our study is the only prospective cohort study in India that i) followed a cohort during the Omicron-I and Omicron-II periods and ii) provided insight into the transmission dynamics of specific Omicron sub-lineages. Given the declining testing rates and sequencing for SARS-CoV-2 in India after the Omicron wave, our data on incident infections during the Omicron-II period are particularly relevant and provide valuable information for public health measures, such as booster recommendations and targeting vulnerable populations. Additionally, the study collected data on antibody kinetics over 2 years, allowing us to assess the role of antigen imprinting following multiple infections and vaccinations to understand potential future clinical outcomes, which is subject to further analysis.

However, this study has certain limitations. First, data collected was delayed after the second pandemic wave in India, which may have led to missed opportunities to analyze real-time data trends during its peak. Second, we were unable to assess vaccine effectiveness due to the lack of blood sample collection at specific time points following vaccination. Third, all RT-PCR-positive samples were incomplete sequenced due to resource constraints, and RT-PCR data on prior exposure could not be collected, although baseline serology served as a valuable alternative. Thus, the implementation of a structured approach to blood sample collection at specific intervals after vaccination could provide clearer insights into the effectiveness of vaccination. Additionally, potential misclassification of participants based on serology could not be fully controlled.

In conclusion, the cohort experienced the highest rate of SARS-CoV-2 infections during the Omicron-I period. By the end of the study, only 1% of the entire cohort had not been exposed to SARS-CoV-2. This study highlights the efficacy of non-invasive sampling in promoting participant compliance and accurately identifying infections, as reflected by the patterns of the pandemic observed nationwide.

## Contributors

G. K. and J. J. conceived the trial and contributed to the original protocol. J. S. P., R. M., and D. K. contributed to the project administration and data entry. A. R., K. K, P. D., P. S., A. K., J. J. B., A. K., N. T. G., R. B., S. D. T., A. S., and E. J. performed the saliva processing and RT-PCR. D. P., C. J., and P. J. performed the whole genome sequencing for SARS-CoV-2. S. B., S. I., V. G., and R. M. performed the serological assays and analysis. R. M., J. S. P., I. T., and D. K. performed statistical analysis. R. M. and S. A. K. submitted the sequences to GISAID and performed the phylogenetic analysis. A. G. is a part of the scientific advisory committee for the CORES study. R. M. drafted the initial report. G. K. and J. J. finalized the report. All authors reviewed and approved the final report.

## Data sharing statement

De-identified data will be shared upon request to the corresponding author.

## Declaration of interests

We declare no competing interests.
