# Supplementary material for: SARS-CoV-2 infections before, during, and after the Omicron wave: a 2-year Indian community cohort study
Source: Lancet Reg Health Southeast Asia. 2024 Aug 21;28:100470. doi: 10.1016/j.lansea.2024.100470 (PMC11388673; doi:10.1016/j.lansea.2024.100470)
Supplement: Supplementary Tables [file mmc1.docx]

**Supplementary Tables**

**Supplementary Table 1.**

**Supplementary Table 2.**

**Supplementary Table 3.**

**Supplementary Table 4.**

**Supplementary Table 5.**

**Supplementary Table 6.**

**Supplementary Table 7.**

**Supplementary Table 8.**

**Supplementary Table 1. Flow chart depicting the number of sequenced samples subjected to phylogenetic analysis**

| SUBJECTED TO SEQUENCING (n=151) |  |
| --- | --- |
| \|  \| \| --- \| | ID MISMATCH (n=1) |
| SEQUENCE ANALYSED (n=150) |  |
| \|  \| \| --- \| | FAILED (n=11) |
| SEQUENCE QUALITY SCREENED (n=139) |  |
| \|  \| \| --- \| | COVERAGE BELOW 80 (n=22) |
| FINAL SEQUENCES FOR PHYLOGENETICS (n=117) |  |
|  |  |
| COVERAGE >=90% | 102 |
| COVERAGE 80-89% | 15 |
| NUMBER OF REFERENCE SEQUENCES | 561 |

**Supplementary Table 2. Accession numbers for the whole genome sequenced strains submitted in the GSAID platform**

| **Sequence ID** | **GISAID clade** | **Variant** | **PANGO lineage** | **Lineage** | **LAB ID** | **Date of collection** | **GISAID accession** |
| --- | --- | --- | --- | --- | --- | --- | --- |
| SEQ_15107 | 21L | Omicron | BA.2.10 | BA.2.10 | CORES-S-25031 | January 10, 2022 | EPI_ISL_16741140 |
| SEQ_20562 | 21L | Omicron | BA.2.10 | BA.2.10 | CORES-S-25525 | January 10, 2022 | EPI_ISL_16741139 |
| SEQ_20557 | 21L | Omicron | BA.2.10 | BA.2.10 | CORES-S-25671 | January 10, 2022 | EPI_ISL_16741138 |
| SEQ_20558 | 21K | Omicron | BA.1 | BA.1 | CORES-S-25940 | January 10, 2022 | EPI_ISL_16741137 |
| SEQ_16794 | 21K | Omicron | B.1.1.7 | B.1.1.7 | CORES-S-26392 | January 10, 2022 | EPI_ISL_16741136 |
| SEQ_19510 | 21L | Omicron | BA.2.10 | BA.2.10 | CORES-S-26529 | January 11, 2022 | EPI_ISL_16741135 |
| SEQ_19629 | 21L | Omicron | BA.2.10 | BA.2.10 | CORES-S-26677 | January 17, 2022 | EPI_ISL_16741134 |
| SEQ_19517 | 21L | Omicron | BA.2.10 | BA.2.10 | CORES-S-26846 | January 17, 2022 | EPI_ISL_16741133 |
| SEQ_16795 | 21L | Omicron | BA.2.10 | BA.2.10 | CORES-S-26880 | January 17, 2022 | EPI_ISL_16741182 |
| SEQ_19515 | 22D | Omicron | BA.2 | BA.2 | CORES-S-26953 | January 17, 2022 | EPI_ISL_16741132 |
| SEQ_19506 | 21L | Omicron | BA.2.10 | BA.2.10 | CORES-S-27318 | January 17, 2022 | EPI_ISL_16741131 |
| SEQ_19527 | 21K | Omicron | BA.1 | BA.1 | CORES-S-27404 | January 18, 2022 | EPI_ISL_16741130 |
| SEQ_20554 | 21L | Omicron | BA.2 | BA.2 | CORES-S-27873 | January 24, 2022 | EPI_ISL_16741129 |
| SEQ_15032 | 21L | Omicron | BA.2 | BA.2 | CORES-S-41444 | April 18, 2022 | EPI_ISL_16724090 |
| SEQ_19240 | 21L | Omicron | BA.2.76 | BA.2.76 | CORES-S-51732 | June 20, 2022 | EPI_ISL_16741124 |
| SEQ_19230 | 22D | Omicron | BM.4.1.1 | BA.2.75.3.4.1. | CORES-S-60994 | August 22, 2022 | EPI_ISL_16741126 |
| SEQ_19204 | 22B | Omicron | BA.5.2 | BA.5.2 | CORES-S-62059 | August 29, 2022 | EPI_ISL_16741122 |
| SEQ_19209 | 22D | Omicron | BL.1 | BA.2.75.1.1 | CORES-S-62469 | August 29, 2022 | EPI_ISL_16741117 |
| SEQ_19214 | 22D | Omicron | BM.1.1.3 | BA.2.75.3.1.1 | CORES-S-62913 | August 29, 2022 | EPI_ISL_16741119 |
| SEQ_19215 | 22D | Omicron | BY.1 | BA.2.75.6.1 | CORES-S-63022 | August 30, 2022 | EPI_ISL_16741128 |
| SEQ_20564 | 22D | Omicron | BM.1.1.3 | BA.2.75.3.1.1 | CORES-S-63212 | September 05, 2022 | EPI_ISL_16741125 |
| SEQ_20566 | 22D | Omicron | BA.2.75.6 | BA.2.75.6 | CORES-S-63377 | September 05, 2022 | EPI_ISL_16741118 |
| SEQ_20568 | 22D | Omicron | BL.4 | BA.2.75.1.4 | CORES-S-63658 | September 05, 2022 | EPI_ISL_16741123 |
| SEQ_19216 | 22D | Omicron | BL.2.1 | BA.2.75.1.2.1 | CORES-S-64166 | September 12, 2022 | EPI_ISL_16741120 |
| SEQ_19226 | 22D | Omicron | BM.1.1.3 | BA.2.75.3.1.1 | CORES-S-65024 | September 12, 2022 | EPI_ISL_16741121 |
| SEQ_19227 | 22D | Omicron | BA.2.75 | BA.2.75 | CORES-S-65160 | September 13, 2022 | EPI_ISL_16741127 |
| 89754 | 22F | Omicron | XBB.2.3 | XBB.2.3 | CORES-S-89755 | February 23, 2023 | EPI_ISL_17372253 |
| 89416 | 22F | Omicron | XBB.1 | XBB.1 | CORES-S-89417 | February 24, 2023 | EPI_ISL_17372252 |
| SEQ_8959 | 21J | Omicron | B.1.617.2 | B.1.617.2 | CORES-S-14144 | November 01, 2021 | EPI_ISL_16724091 |
| SEQ_8960 | 21J | Omicron | AY.127 | B.1.617.2.127 | CORES-S-14207 | November 01, 2021 | EPI_ISL_16741180 |
| SEQ_14570 | 21K | Omicron | B.1.1.7 | B.1.1.7 | CORES-S-23161 | December 23, 2021 | EPI_ISL_16724084 |
| SEQ_15102 | 21K | Omicron | B.1.1.7 | B.1.1.7 | CORES-S-23302 | December 27, 2021 | EPI_ISL_16741116 |
| SEQ_15104 | 21L | Omicron | BA.2.10 | BA.2.10 | CORES-S-23856 | December 27, 2021 | EPI_ISL_16741164 |
| SEQ_15105 | Recombinant | Recombinant | XM | XM | CORES-S-24169 | December 27, 2021 | EPI_ISL_16741165 |
| SEQ_14421 | 21L | Omicron | BA.2.10 | BA.2.10 | CORES-S-24740 | January 03, 2022 | EPI_ISL_16724088 |
| SEQ_14422 | 21L | Omicron | BA.2.10 | BA.2.10 | CORES-S-24774 | January 03, 2022 | EPI_ISL_16741177 |
| SEQ_14423 | 21L | Omicron | BA.2.10 | BA.2.10 | CORES-S-24780 | January 03, 2022 | EPI_ISL_16741178 |
| SEQ_14425 | 21L | Omicron | BA.2.10 | BA.2.10 | CORES-S-24933 | January 03, 2022 | EPI_ISL_16724089 |
| SEQ_14426 | 21L | Omicron | BA.2.10 | BA.2.10 | CORES-S-24939 | January 03, 2022 | EPI_ISL_16741179 |
| SEQ_15108 | 21L | Omicron | BA.2.10 | BA.2.10 | CORES-S-25085 | January 03, 2022 | EPI_ISL_16741166 |
| SEQ_15109 | 21L | Omicron | BA.2.10 | BA.2.10 | CORES-S-25095 | January 03, 2022 | EPI_ISL_16741167 |
| SEQ_20561 | 21K | Omicron | B.1.1.7 | B.1.1.7 | CORES-S-25436 | January 03, 2022 | EPI_ISL_16741163 |
| SEQ_20556 | 21L | Omicron | BA.2.10 | BA.2.10 | CORES-S-25557 | January 10, 2022 | EPI_ISL_16741160 |
| SEQ_19520 | 21L | Omicron | BA.2.10 | BA.2.10 | CORES-S-25578 | January 10, 2022 | EPI_ISL_16741143 |
| SEQ_19521 | 21L | Omicron | BA.2.10 | BA.2.10 | CORES-S-25656 | January 10, 2022 | EPI_ISL_16741173 |
| SEQ_20559 | 21L | Omicron | BA.2.10 | BA.2.10 | CORES-S-26044 | January 10, 2022 | EPI_ISL_16741161 |
| SEQ_20560 | 21L | Omicron | BA.2.10 | BA.2.10 | CORES-S-26072 | January 10, 2022 | EPI_ISL_16741162 |
| SEQ_19511 | 21L | Omicron | BA.2.10 | BA.2.10 | CORES-S-26165 | January 10, 2022 | EPI_ISL_16741169 |
| SEQ_19244 | 21M | Omicron | B.1.1.529 | B.1.1.529 | CORES-S-26204 | January 10, 2022 | EPI_ISL_16741142 |
| SEQ_16791 | 21L | Omicron | BA.2.10 | BA.2.10 | CORES-S-26430 | January 10, 2022 | EPI_ISL_16741181 |
| SEQ_19628 | 21L | Omicron | BA.2.10 | BA.2.10 | CORES-S-26668 | January 17, 2022 | EPI_ISL_16741175 |
| SEQ_15113 | 21L | Omicron | BA.2.10 | BA.2.10 | CORES-S-26796 | January 17, 2022 | EPI_ISL_16741168 |
| SEQ_19516 | 21L | Omicron | BA.2 | BA.2 | CORES-S-26859 | January 17, 2022 | EPI_ISL_16741171 |
| SEQ_19514 | 21L | Omicron | BA.2.10 | BA.2.10 | CORES-S-26928 | January 17, 2022 | EPI_ISL_16741170 |
| SEQ_19630 | 21L | Omicron | BA.2.10 | BA.2.10 | CORES-S-26968 | January 17, 2022 | EPI_ISL_16741176 |
| SEQ_19518 | 21L | Omicron | BA.2.10 | BA.2.10 | CORES-S-27051 | January 17, 2022 | EPI_ISL_16741172 |
| SEQ_19237 | 22D | Omicron | BA.2.75.3 | BA.2.75.3 | CORES-S-49969 | June 16, 2022 | EPI_ISL_16741196 |
| SEQ_19238 | 21L | Omicron | BA.2 | BA.2 | CORES-S-51499 | June 20, 2022 | EPI_ISL_16741197 |
| SEQ_19239 | 21L | Omicron | BA.2 | BA.2 | CORES-S-51533 | June 20, 2022 | EPI_ISL_16741198 |
| SEQ_17498 | 22B | Omicron | BA.5.2 | BA.5.2 | CORES-S-54221 | July 11, 2022 | EPI_ISL_16741144 |
| SEQ_17484 | 21L | Omicron | BA.2.76 | BA.2.76 | CORES-S-54576 | July 11, 2022 | EPI_ISL_16741145 |
| SEQ_17485 | 22B | Omicron | BF.3 | BA.5.2.1.3 | CORES-S-54663 | July 11, 2022 | EPI_ISL_16741146 |
| SEQ_17486 | 22B | Omicron | BE.4 | BA.5.3.1.4 | CORES-S-54676 | July 11, 2022 | EPI_ISL_16741147 |
| SEQ_17478 | 22B | Omicron | BF.23 | BA.5.2.1.23 | CORES-S-54839 | July 11, 2022 | EPI_ISL_16741148 |
| SEQ_17479 | 22B | Omicron | BF.3 | BA.5.2.1.3 | CORES-S-54861 | July 11, 2022 | EPI_ISL_16724085 |
| SEQ_17480 | 22B | Omicron | BF.3 | BA.5.2.1.3 | CORES-S-55061 | July 11, 2022 | EPI_ISL_16741149 |
| SEQ_17472 | 22D | Omicron | BA.2.75.3 | BA.2.75.3 | CORES-S-55493 | July 18, 2022 | EPI_ISL_16741150 |
| SEQ_17473 | 22B | Omicron | BA.5.2.1 | BA.5.2.1 | CORES-S-55541 | July 18, 2022 | EPI_ISL_16724086 |
| SEQ_17474 | 22B | Omicron | BF.3 | BA.5.2.1.3 | CORES-S-55556 | July 18, 2022 | EPI_ISL_16741151 |
| SEQ_17475 | 22B | Omicron | BF.3 | BA.5.2.1.3 | CORES-S-55723 | July 18, 2022 | EPI_ISL_16741152 |
| SEQ_17476 | 21L | Omicron | BA.2.10 | BA.2.10 | CORES-S-55918 | July 18, 2022 | EPI_ISL_16741153 |
| SEQ_17499 | 22B | Omicron | BA.5.2 | BA.5.2 | CORES-S-58684 | August 08, 2022 | EPI_ISL_16741154 |
| SEQ_17500 | 22D | Omicron | BA.2.75.3 | BA.2.75.3 | CORES-S-58705 | August 08, 2022 | EPI_ISL_16741155 |
| SEQ_19233 | 22D | Omicron | BA.2.75.6 | BA.2.75.6 | CORES-S-58972 | August 08, 2022 | EPI_ISL_16741156 |
| SEQ_17505 | 22D | Omicron | BA.2.75.6 | BA.2.75.6 | CORES-S-58972 | August 08, 2022 | PI_ISL_16741193 |
| SEQ_17490 | 22B | Omicron | BF.3 | BA.5.2.1.3 | CORES-S-58978 | August 08, 2022 | EPI_ISL_16741157 |
| SEQ_19234 | 22D | Omicron | BA.2.75.1 | BA.2.75.1 | CORES-S-59363 | August 08, 2022 | EPI_ISL_16741194 |
| SEQ_17494 | 22D | Omicron | BA.2.75.1 | BA.2.75.1 | CORES-S-59443 | August 08, 2022 | EPI_ISL_16741141 |
| SEQ_17495 | 22B | Omicron | BF.3 | BA.5.2.1.3 | CORES-S-59520 | August 08, 2022 | EPI_ISL_16741158 |
| SEQ_19236 | 21L | Omicron | BA.2.76 | BA.2.76 | CORES-S-59612 | August 08, 2022 | EPI_ISL_16741159 |
| SEQ_17496 | 21L | Omicron | BA.2.76 | BA.2.76 | CORES-S-59612 | August 08, 2022 | EPI_ISL_16741195 |
| SEQ_19231 | 22D | Omicron | BL.1 | BA.2.75.1.1 | CORES-S-61063 | August 22, 2022 | EPI_ISL_16741192 |
| SEQ_19206 | 22D | Omicron | BY.1 | BA.2.75.6.1 | CORES-S-62172 | August 29, 2022 | EPI_ISL_16741183 |
| SEQ_19207 | 22D | Omicron | BA.2.75.5 | BA.2.75.5 | CORES-S-62242 | August 29, 2022 | EPI_ISL_16741184 |
| SEQ_19210 | 22D | Omicron | BA.2.75.10 | BA.2.75.10 | CORES-S-62477 | August 29, 2022 | EPI_ISL_16741185 |
| SEQ_19211 | 22D | Omicron | BL.3 | BA.2.75.1.3 | CORES-S-62712 | August 29, 2022 | EPI_ISL_16741186 |
| SEQ_20563 | 22B | Omicron | BA.5.2 | BA.5.2 | CORES-S-63095 | September 05, 2022 | EPI_ISL_16724087 |
| SEQ_19217 | 22D | Omicron | BN.1.4 | BA.2.75.5.1.4 | CORES-S-64479 | September 12, 2022 | EPI_ISL_16741187 |
| SEQ_19219 | 22D | Omicron | BL.1 | BA.2.75.1.1 | CORES-S-64622 | September 12, 2022 | EPI_ISL_16741188 |
| SEQ_19224 | 22D | Omicron | BY.1 | BA.2.75.6.1 | CORES-S-64817 | September 12, 2022 | EPI_ISL_16741189 |
| SEQ_19228 | 22D | Omicron | BY.1 | BA.2.75.6.1 | CORES-S-65194 | September 13, 2022 | EPI_ISL_16741190 |
| SEQ_19229 | 22D | Omicron | BA.2.75 | BA.2.75 | CORES-S-65203 | September 13, 2022 | EPI_ISL_16741191 |
| SEQ_19532 | 22F | Omicron | XBB.1 | XBB.1 | CORES-S-66249 | September 19, 2022 | EPI_ISL_16741174 |
| SEQ_88688 | 22F | Omicron | XBB.1 | XBB.1 | CORES-S-88689 | February 17, 2023 | EPI_ISL_17372262 |
| SEQ_89084 | 22F | Omicron | XBB.1 | XBB.1 | CORES-S-89085 | February 24, 2023 | EPI_ISL_17372263 |
| SEQ_89140 | 22F | Omicron | XBB.2.3 | XBB.2.3 | CORES-S-89141 | February 24, 2023 | EPI_ISL_17372264 |
| SEQ_89940 | 22F | Omicron | XBB.2.3 | XBB.2.3 | CORES-S-89941 | February 24, 2023 | EPI_ISL_17372265 |
| SEQ_89952 | 22F | Omicron | XBB.1 | XBB.1 | CORES-S-89953 | February 24, 2023 | EPI_ISL_17372266 |
| SEQ_65316 | 22D | Omicron | BL.2 | BA.2.75.1.2 | CORES-S-65317 | September 19, 2022 | EPI_ISL_17372254 |
| SEQ_65509 | 22D | Omicron | BA.2.75.10 | BA.2.75.10 | CORES-S-65510 | September 19, 2022 | EPI_ISL_17375706 |
| SEQ_65539 | 22F | Omicron | XBB.3 | XBB.3 | CORES-S-65540 | September 19, 2022 | EPI_ISL_17372255 |
| SEQ_65769 | 22D | Omicron | BM.1.1.3 | BA.2.75.3.1.1.3 | CORES-S-65770 | September 19, 2022 | EPI_ISL_17372257 |
| SEQ_65789 | 22D | Omicron | BL.1 | BA.2.75.1.1 | CORES-S-65790 | September 19, 2022 | EPI_ISL_17372256 |
| SEQ_65792 | 22D | Omicron | BY.1 | BA.2.75.6.1 | CORES-S-65793 | September 19, 2022 | EPI_ISL_17372258 |
| SEQ_65970 | 22D | Omicron | BM.1.1.1 | BA.2.75.3.1.1.1 | CORES-S-65971 | September 19, 2022 | EPI_ISL_17372259 |
| SEQ_66102 | 22D | Omicron | BA.2.75.5 | BA.2.75.5 | CORES-S-66103 | September 19, 2022 | EPI_ISL_17372260 |
| SEQ_66103 | 22D | Omicron | BA.2.75.2 | BA.2.75.2 | CORES-S-66104 | September 19, 2022 | EPI_ISL_17372261 |
| SEQ_15101 | Recombinant | Omicron | XAB | XAB | CORES-S-23285 | December 27, 2021 | EPI_ISL_17375705 |
| SEQ_15103 | 21L | Omicron | BA.2.10 | BA.2.10 | CORES-S-23337 | December 27, 2021 | EPI_ISL_17375704 |
| SEQ_15106 | 21L | Omicron | BA.2 | BA.2 | CORES-S-24357 | January 03, 2022 | EPI_ISL_17375703 |
| SEQ_19507 | 21L | Omicron | BA.2.10 | BA.2.10 | CORES-S-27487 | January 24, 2022 | EPI_ISL_17375702 |
| SEQ_17481 | 22B | Omicron | BF.3 | BA.5.2.1.3 | CORES-S-54363 | July 11, 2022 | EPI_ISL_17375701 |
| SEQ_19235 | 22D | Omicron | BA.2.75.1 | BA.2.75.1 | CORES-S-59443 | August 8, 2022 | EPI_ISL_16741141 |
| SEQ_19212 | 22D | Omicron | BM.1.1.3 | BA.2.75.5.1.1. | CORES-S-62816 | August 29, 2022 | EPI_ISL_17375700 |
| SEQ_19213 | 22D | Omicron | BM.1.1.3 | BA.2.75.3.1.1 | CORES-S-62832 | August 29, 2022 | EPI_ISL_17375699 |
| SEQ_20567 | 22D | Omicron | BA.2.75.1 | BA.2.75.1 | CORES-S-63418 | September 05, 2022 | EPI_ISL_17375698 |
| SEQ_19223 | 22D | Omicron | BA.2.75.10 | BA.2.75.10 | CORES-S-64784 | September 12, 2022 | EPI_ISL_17375697 |

**Supplementary Table 3. Cause of death among the study participants during the two-year follow-up**

| Total deaths in the cohort | 22 |
| --- | --- |
| COVID-19 related deaths | 0 |
| Acute coronary syndrome | 7 |
| Acute respiratory illness (pneumonia) | 2 |
| Cerebrovascular accident | 1 |
| Neoplasm | 1 |
| Road traffic injury | 1 |
| Undetermined | 3 |
| Suicide | 2 |
| No response from the participant's relatives | 3 |

**Supplementary Table 4. Vaccination status and the type of vaccines received by the participants of the CORES study**

| **Vaccination status** | **No** | 259 (22·2%) |
| --- | --- | --- |
|  | **Yes** | 907 (77·8%) |
| **Vaccine type** | **Covaxin** | 212 (23·4%) |
|  | **Covishield** | 695 (76·6%) |
| **Vaccine dosage** | **One dose** | 312 (34·4%) |
|  | **Two doses** | 554 (61·1%) |
|  | **Three doses** | 41 (4·5%) |

**Supplementary Table 5. Incidence rates based on the antibody levels across each time period**

**Baseline serology: (n=1229)**

| **Period** | **Group** | **Total cases** | **PYO** | **Incidence rate** |
| --- | --- | --- | --- | --- |
| **Pre-Omicron** | **less_25 (<3712·92)** | 23 | 97·07 | 236·9 (150·2,355·53) |
|  | **25_to_75 (3712·92-82058·88)** | 18 | 215·2 | 83·6 (49·57,132·19) |
|  | **above_75 (>82058·88)** | 15 | 125·09 | 119·9 (67·11,197·78) |
| **Omicron-I** | **less_25 (<3712·92)** | 25 | 2·97 | 8415·9 (5446·33,12423·53) |
|  | **25_to_75 (3712·92-82058·88)** | 33 | 5·56 | 5937·6 (4087·14,8338·54) |
|  | **above_75 (>82058·88)** | 5 | 1·44 | 3478·6 (1129·48,8117·83) |
| **Overall** |  | **119** | **447·33** | **266·02 (220·38,318·34)** |

**Sixth month serology: (n=1166)**

| **Period** | **Group** | **Total cases** | **PYO** | **Incidence rate** |
| --- | --- | --- | --- | --- |
| **Pre-Omicron** | **less_25 (<11063·77)** | 1 | 2·78 | 359·8 (9·11,2004·97) |
|  | **25_to_75 (11063·77-155168·44)** | 1 | 5·94 | 168·3 (4·26,937·81) |
|  | **above_75 (>155168·44)** | 0 | 2·8 | 0 (0,1318·36) |
| **Omicron-I** | **less_25 (<11063·77)** | 152 | 94·9 | 1601·6 (1357·11,1877·42) |
|  | **25_to_75 (11063·77-155168·44)** | 277 | 204·03 | 1357·6 (1202·45,1527·3) |
|  | **above_75 (>155168·44)** | 106 | 110·24 | 961·6 (787·25,1162·99) |
| **Omicron-II** | **less_25 (<11063·77)** | 15 | 48·53 | 309·1 (173·01,509·84) |
|  | **25_to_75 (11063·77-155168·44)** | 22 | 75·15 | 292·8 (183·47,443·25) |
|  | **above_75 (>155168·44)** | 16 | 34·27 | 466·9 (266·89,758·25) |
| **Overall** |  | **590** | **578·63** | **1019·7 (939·03,1105·35)** |

**Twelfth month serology: (n=1099)**

| **Period** | **Group** | **Total cases** | **PYO** | **Incidence rate** |
| --- | --- | --- | --- | --- |
| **Omicron-I** | **less_25 (<38552·23)** | 0 | 2·22 |  |
|  | **25_to_75 (38552·23-170741·83)** | 0 | 4·41 |  |
|  | **above_75 (>170741·83)** | 0 | 1·57 |  |
| **Omicron-II** | **less_25 (<38552·23)** | 172 | 279·37 | 615·7 (527·1,714·88) |
|  | **25_to_75 (38552·23-170741·83)** | 304 | 550·32 | 552·4 (492·05,618·12) |
|  | **above_75 (>170741·83)** | 117 | 276·86 | 422·6 (349·5,506·48) |
| **Overall** |  | **593** | **1114·8** | **531·9 (490,576·55)** |

***PYO-Person Years of Observation. The antibody levels were stratified based on the antibody levels (<25^th^ centile, 25^th^-75^th^ centile and <75^th^ centile)***

**Supplementary Table 6. Age and gender-adjusted risk ratio among seronegative individuals as compared to seropositive individuals across the three time periods**

| **Time Periods** | **Pre -Omicron** | | **Omicron-I** | | **Omicron-II** | |
| --- | --- | --- | --- | --- | --- | --- |
| **Serology status** | **Seronegative** | **Seropositive** | **Seronegative** | **Seropositive** | **Seronegative** | **Seropositive** |
| **Total cases** | 15 | 36 | 58 | 548 | 14 | 633 |
| **PYO** | 7051 | 378·4 | 33·8 | 393·5 | 44·2 | 1284·7 |
| **Incidence rate** | 212·7 (119·1,350·9) | 95·1 (6·6,131·7) | 1714·6 (1302·01,2216·5) | 1392·6 (1278·4,1514·2) | 316·51(173·04,531·05) | 492·7 (455·1,532·6) |
| **RR** | 2·2 (1·19,4) | ref | 1·23 (0·9,1·6) | ref | 0·64 (0·3,1·1) | ref |
| **Adj.RR_vacc** | 2·3 (1·2,4·2) | ref | 1·15 (0·9,1·5) | ref | 0·65 (0·3,1·1) | ref |
| **Adj.RR_vacc+age** | 2·32 (1·2,4·2) | ref | 1·13 (0·8,1·47) | ref | 0·64 (0·3,1) | ref |
| **Adj.RR_vacc+gender** | 2·32 (1·2,4·2) | ref | 1·15 (0·8,1·5) | ref | 0·63 (0·3,1) | ref |
| **Adj.RR_age** | 2·21 (1·2,3·9) | ref | 1·2 (0·9,1·55) | ref | 0·62 (0·3,1) | ref |
| **Adj.RR_gender** | 2·22 (1·2,3·9) | ref | 1·2 (0·9,1·59) | ref | 0·62 (0·3,1) | ref |
| **Adj.RR_age+gender** | 2·2 (1·2,3·9) | ref | 1·2 (0·9,1·56) | ref | 0·61 (0·3,0·9) | ref |
| **Adj.RR_vac+age+gender** | 2·3 (1·2,4·2) | ref | 1·13 (0·8,1·48) | ref | 0·62 (0·3,1) | ref |

***PYO- Person years of Observation, RR- Risk Ratio, vacc- vaccination, Adj- Adjusted***

**Supplementary Table 7: Age and gender-adjusted risk ratio among unvaccinated individuals as compared to vaccinated individuals across the three time periods**

| **Time Periods** | **Pre -Omicron** | | **Omicron-I** | | **Omicron-II** | |
| --- | --- | --- | --- | --- | --- | --- |
| **Vaccination status** | **Unvaccinated** | **Vaccinated** | **Unvaccinated** | **Vaccinated** | **Unvaccinated** | **Vaccinated** |
| Total cases | 22 | 29 | 162 | 444 | 140 | 507 |
| Pyo | 218·23 | 230·65 | 95·63 | 331·71 | 305·51 | 1023·47 |
| Incidence rate | 100·8 (63·2,152·6) | 125·73(84·2,180·57) | 1693·9 (1443·16,1975·85) | 1338·5 (1216·9,1469) | 458·2 (385·5,540·8) | 495·4 (453·2,540·4) |
| RR | 0·8 (0·4,1·4) | ref | 1·2 (1·06,1·5) | ref | 0·9 (0·7,1·1) | ref |
| Adj·RR_vacc | 0·7 (0·4,1·3) | ref | 1·2 (1,1·5) | ref | 0·9 (0·7,1·1) | ref |
| Adj.RR_vacc+age | 0·7 (0·4,1·3) | ref | 1·2 (1,1·5) | ref | 0·9 (0·7,1·1) | ref |
| Adj.RR_vacc+gender | 0·7 (0·4,1·3) | ref | 1·24 (1,1·5) | ref | 0·9 (0·7,1·1) | ref |
| Adj.RR_age | 0·8 (0·4,1·4) | ref | 1·25 (1·04,1·5) | ref | 0·9 (0·7,1·1) | ref |
| Adj.RR_gender | 0·8 (0·4,1·3) | ref | 1·27 (1·06,1·5) | ref | 0·9 (0·7,1·1) | ref |
| Adj.RR_age+gender | 0·8 (0·4,1·4) | ref | 1·25 (1·04,1·5) | ref | 0·9 (0·7,1·1) | ref |
| Adj. RR_vacc+age+gender | 0·7 (0·4,1·3) | ref | 1·23 (1·02,1·5) | ref | 0·9 (0·7,1·1) | ref |

***PYO- Person years of Observation, RR- Risk Ratio, vacc- vaccination***

**Supplementary Table 8. Incidence rates in the four categories among symptomatic SARS-CoV-2 infections**

| **Time Periods** | **Group** | **Total cases** | **PYO** | **Incidence rate**  **(95% C.I)** | **P-value** |
| --- | --- | --- | --- | --- | --- |
| **Overall** |  | 306 | 2205·2 | 138·7 (123·6 ,155·2) |  |
| **Pre-Omicron** | **SNUV** | 1 | 43·01 | 23·2 (0·6,129·6) | - |
|  | **SNV** | 3 | 27·5 | 109·0 (22·5,318·8) | 0·14 |
|  | **SPUV** | 0 | 175·2 | 0(0,21·1) | 1·00 |
|  | **SPV** | 1 | 203·1 | 4·9 (0·1,27·4) | 0·49 |
| **Omicron-I** | **SNUV** | 14 | 17·1 | 818 (447·2,1372·5) | - |
|  | **SNV** | 8 | 16·7 | 478·7 (206·7,943·2) | 0·55 |
|  | **SPUV** | 45 | 78·5 | 573·1 (418,766·9) | 0·51 |
|  | **SPV** | 122 | 315 | 387·3 (321·6,462·5) | 0·11 |
| **Omicron-II** | **SNUV** | 1 | 24·5 | 40·8 (1·03,227·1) | - |
|  | **SNV** | 2 | 19·7 | 101·5 (12·3,366·7) | 0·24 |
|  | **SPUV** | 24 | 280·9 | 85·4 (54·7,127·1) | 0·60 |
|  | **SPV** | 85 | 1003·7 | 84·6 (67·6,104·7) | 0·66 |

***PYO- Person Years of Observation***
